# Epitaxial lift-off of freestanding (011) and (111) SrRuO_3_ thin films using a water sacrificial layer

**DOI:** 10.1038/s41598-021-91848-2

**Published:** 2021-06-14

**Authors:** Phu T. P. Le, Johan E. ten Elshof, Gertjan Koster

**Affiliations:** MESA+ Institute for Nanotechnology, University of Twente, P.O. Box 217, 7500 AE Enschede, The Netherlands

**Keywords:** Materials science, Nanoscience and technology, Physics

## Abstract

Two-dimensional freestanding thin films of single crystalline oxide perovskites are expected to have great potential in integration of new features to the current Si-based technology. Here, we showed the ability to create freestanding single crystalline (011)- and (111)-oriented SrRuO_3_ thin films using Sr_3_Al_2_O_6_ water-sacrificial layer. The epitaxial Sr_3_Al_2_O_6_(011) and Sr_3_Al_2_O_6_(111) layers were realized on SrTiO_3_(011) and SrTiO_3_(111), respectively. Subsequently, SrRuO_3_ films were epitaxially grown on these sacrificial layers. The freestanding single crystalline SrRuO_3_(011)_pc_ and SrRuO_3_(111)_pc_ films were successfully transferred on Si substrates, demonstrating possibilities to transfer desirable oriented oxide perovskite films on Si and arbitrary substrates.

## Introduction

The integration of transition metal oxide (TMO) thin films and their heterostructures on Si are promising to provide new exciting features in applications of electronics, photonics, sensors, solid state lightning, microelectromechanical systems and so on, because of TMOs’ rich physical properties^1–4^. The introduction of a buffer layer SrTiO_3_(001) (STO) on Si(001) has triggered the development of epitaxial growth of oxide perovskites on Si(001)^5^. Furthermore, efforts have been made to epitaxially grow (La_x_Y_1-x_)_2_O_3_ and Sc_2_O_3_ buffer layers on Si(111)^6,7^. However, the introduction of buffer layers on Si suffers from complexities due to the reduction reaction and interdiffusion between oxides and Si at the interface while retaining the epitaxial relationship^8–10^. Alternatively, oxide nanosheets have been considered as crystalline templates to bridge TMOs and Si^11,12^. Although various TMO thin films have been directed in single out-of-plane orientation using oxide nanosheets^12–14^, true epitaxy has not been achieved over the large scale on Si substrates yet.

Recently, the epitaxial lift-off technique that uses a sacrificial layer has emerged to prepare freestanding films of single crystalline TMOs, which can be transferred onto Si substrates^15–18^. The sacrificial layer acts as a crystallographic template to direct the epitaxial growth of TMO thin films, while it should be selectively removable using a chemical etchant without degrading the properties of the TMO thin films. MgO, La_0.7_Sr_0.3_MnO_3_ and Sr_3_Al_2_O_6_ (SAO) have primarily served as sacrificial layers to prepare freestanding TMO thin films thanks to their removability and epitaxial growth on single crystal substrates^15–17^. In addition to the compatible crystal structure of the oxide perovskites, the SAO sacrificial layer can be etched away using water, reducing contaminants and keeping the high-quality of oxide perovskite thin films^17^.

Various freestanding oxide perovskites with (001) orientation releasing from SAO sacrificial layers have been fabricated and their properties have been studied^19–22^. The other (011) and (111) orientations also offer the ability to control the physical properties of oxide perovskites. For instance, with a thickness of 3 to 12 nm on LaAlO_3_ substrates, La_0.67_Sr_0.33_MnO_3_(001) was insulating while La_0.67_Sr_0.33_MnO_3_ (011) was metallic^23^. The magnetic properties of several manganites were more enhanced in (011) than in (001) orientation^24,25^. The crystal structure of oxide perovskites can be regarded as buckled honeycomb-liked lattices in the [111] direction, which is a prerequisite for accessing many quantum phenomena, for example 2-dimensional (2D) topological insulators and the quantum anomalous Hall state^26–28^. Therefore, freestanding oxide perovskites with (011) and (111) orientations would add new features to Si. Furthermore, the metallic itinerant ferromagnetic SrRuO_3_ (SRO) is a viable starting point for the growth of all oxide heterostructures thanks to its highly chemical and thermal stability^29–32^. In this study, the epitaxial growth of the SAO sacrificial layers on STO(011) and STO(111) orientations were realized using pulsed laser deposition (PLD). Subsequently, SRO films were epitaxially grown on SAO/STO heterostructures. The freestanding films of single crystalline SRO(011)_pc_ and SRO(111)_pc_, where _pc_ stands for pseudocubic, were successfully transferred onto Si substrates. Before the epitaxial lift-off, large magnetic moments of 3.2 μ_B_/Ru^4+^ and 3.5 μ_B_/Ru^4+^ for SRO(011)_pc_ and SRO(111)_pc_ films were observed, suggesting Ru^4+^ in the mixed state of low- and high-spin states and high-spin state, respectively. In contrast, the transferred SRO(011)_pc_ SRO(111)_pc_ films showed a magnetic moment of 1.0 μ_B_/Ru^4+^ and 1.7 μ_B_/Ru^4+^, respectively, which resulted from the low-spin state of Ru^4+^.

## Results and discussion

**Figure 1:**
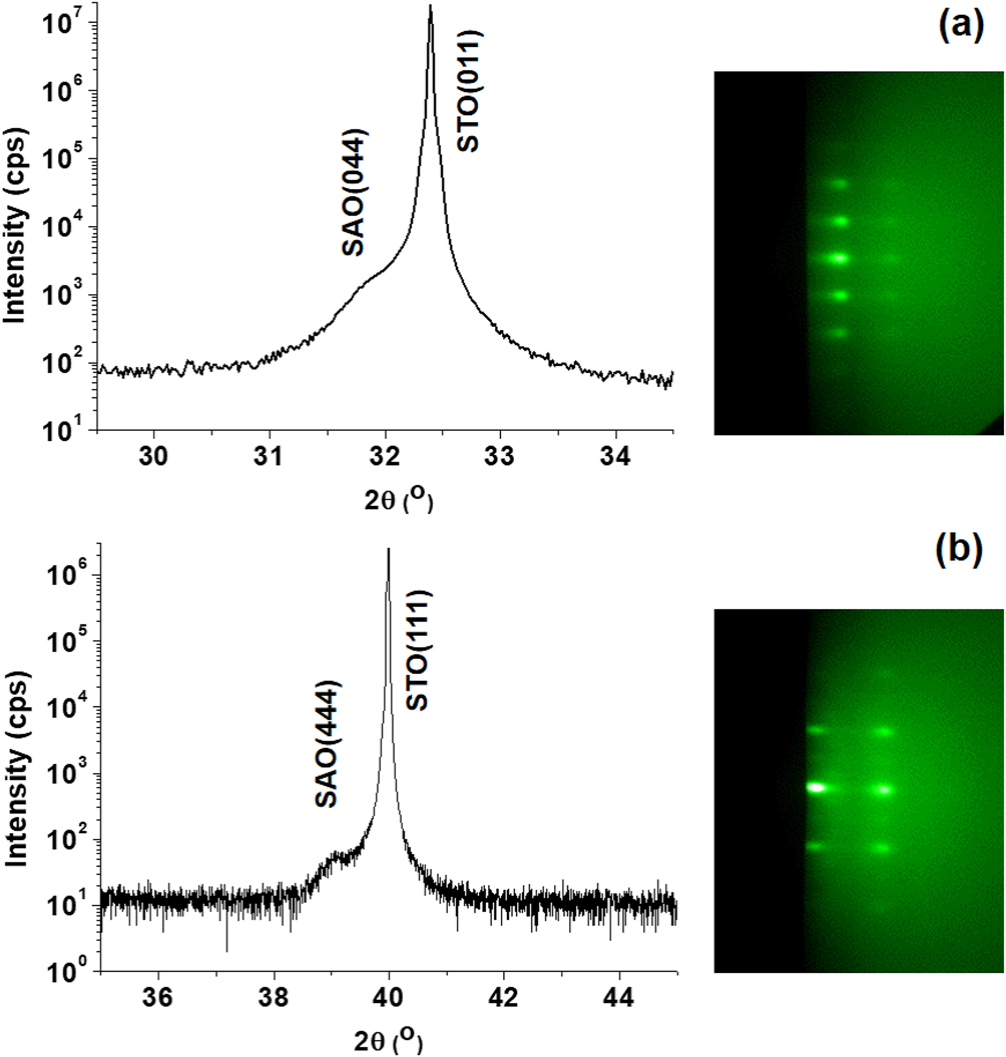
Epitaxial growth of SAO on STO(011) and STO(111) substrates. Panel (**a**) and (**b**) show XRD patterns of 100 nm SAO layers on STO(011) and STO(111) substrates, respectively, with capping STO layers. On the right-hand side, the spotty RHEED patterns of SAO layers were recorded along the [01-1] direction.

Figure 1 shows X-ray diffraction (XRD) patterns of 100 nm SAO films on STO(011) and STO(111) substrates and the corresponding reflection high-energy electron diffraction (RHEED) patterns of the SAO films (see Supplementary Figure S1 for the full scan XRD from 20° to 90°). The peak positions of SAO films at 31.87° and 39.10° matched with the bulk values of the SAO(044) and SAO(444) reflections, respectively, and the clear spotty RHEED patterns, which were recorded along the [01-1] direction of STO(011) and STO(111), indicated single crystalline nature of SAO films. The crystal structure of SAO has been described as a superstructure of 64 cubic perovskite units, in which the unit formula ABO_3_ is (Sr_7/8_□_1/8_)(Sr_1/4_Al_3/4_)(O_3/4_□_1/4_)_3_ with vacancies at A and O positions^33^. Taking domain matching and the 2D symmetry of the crystal planes between SAO and STO into account, the epitaxial growth of SAO(011) and SAO(111) can be realized on STO(011) and STO(111), respectively, with a lattice mismatch of 1.43%.

**Figure 2:**
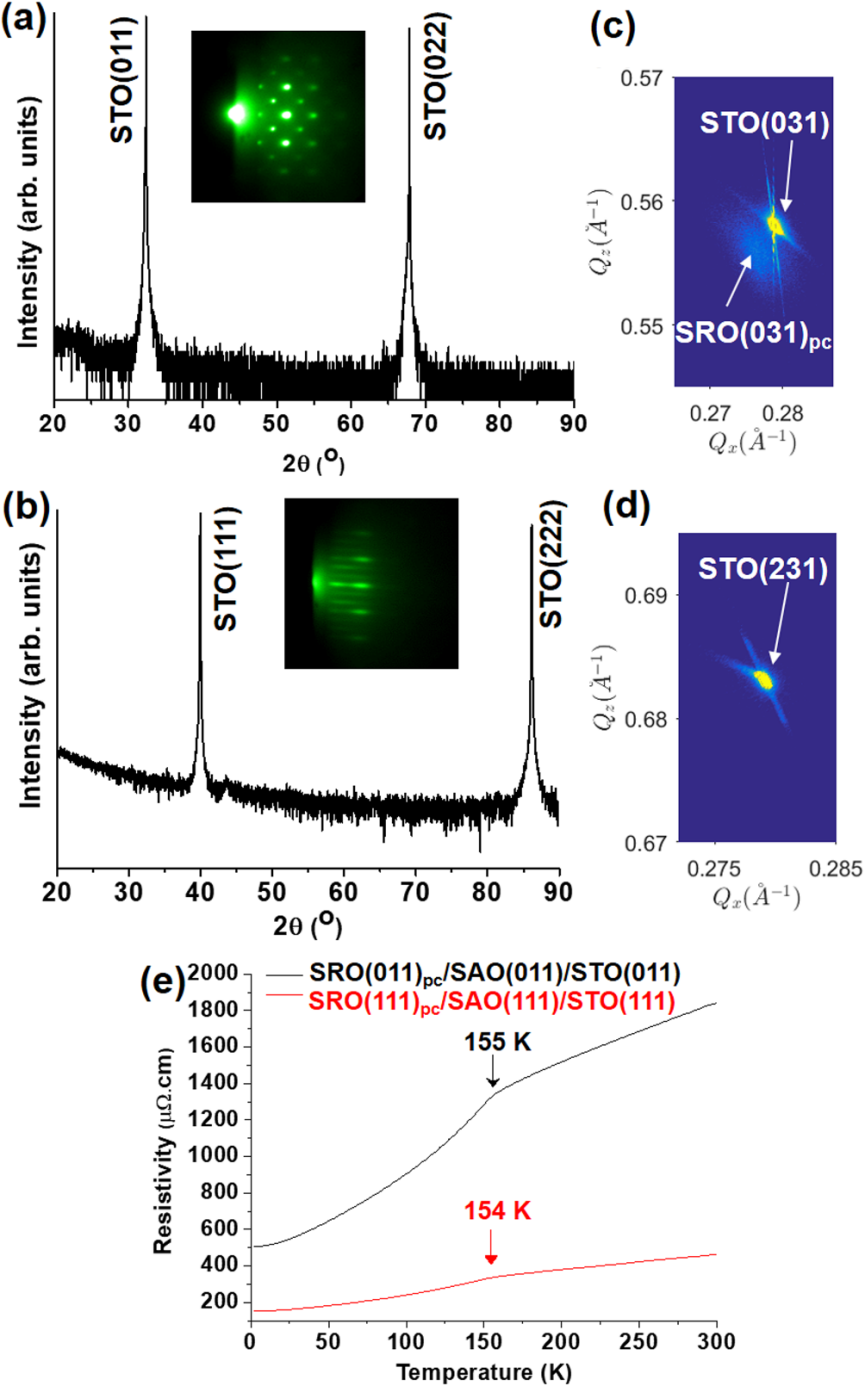
Identification of SRO films on sacrificial SAO layers. Panel (**a**) and (**b**) show that the XRD patterns of SRO(011)_pc_/SAO(011)/STO(011) and SRO(111)_pc_/SAO(111)/STO(111), respectively, could not resolve the reflections of SRO and SAO probably because the their reflections were close to those of STO and their intensities were much weaker than that of STO. The insets show clear spotty and streaky RHEED patterns, which were taken along the [01-1] direction of SRO(011)_pc_ and SRO(111)_pc_ films, respectively, indicating well-crystallized single phase SRO films. RSM around the STO(031) reflection (**c**) indicated that the SRO(011)_pc_ film was not fully strained by the STO(011) substrate, while RSM around the STO(231) reflection (**d**) did not resolve the SRO(231) reflection of the SRO(111)_pc_ film. The transport measurements (**e**) of SRO(011)_pc_/SAO(011)/STO(011) and SRO(111)_pc_/SAO(111)/STO(111) exhibited characteristic behavior of SRO films.

In Fig. 2a and b, the 2θ-ω XRD patterns of SRO grown on SAO(011) and SAO(111), respectively, only showed peaks originating from the STO substrates. SAO(044) and SAO(444) peaks were not resolved in these XRD patterns probably because the SAO layers were thin, about 9.4 nm. However, the spotty and streaky RHEED patterns, which were recorded along the [01-1] direction of SRO grown on SAO(011) and SAO(111), indicated well-crystallized SRO films. It is worth mentioning that SRO films were directly grown on STO(011) and STO(111) substrates under the same growth condition of SRO layers in SRO/SAO/STO samples, SRO(011)_pc_ and SRO(111)_pc_ reflections were clearly observed (see Supplementary Figure S2). With regard to SRO(011)_pc_/SAO(011)/STO(011), the reciprocal space map (RSM) around the STO(031) reflection (Fig. 2c) showed that SRO(011)_pc_ was not fully strained to the STO(011) substrate. The SRO(011)_pc_ film had d_(011)_ = 2.768 Å, which was close to d_(011)_ = 2.762 Å of the STO substrate, and that was why the SRO(011)_pc_ peaks were not observed in Fig. 2a. Meanwhile, the RSM of SRO(111)_pc_/SAO(111)/STO(111) (Fig. 2d) only showed a STO(231) peak. The SRO(111)_pc_ reflections were not resolved probably because its (111) reflections were too close to those of STO(111) due to the insertion of the SAO(111) layer and its peak intensity was quite low compared to that of STO(111). The temperature-dependent resistivities of SRO(011)_pc_/SAO(011)/STO(011) and SRO(111)_pc_/SAO(111)/STO(111) are shown Fig. 2e. Both samples exhibited metallic behavior with a typical kink, which is caused by the ferromagnetic transition of SRO. The transition temperatures T_c_ were 155 K and 154 K for SRO(011)_pc_/SAO(011)/STO(011) and SRO(111)_pc_/SAO(111)/STO(111), respectively, which are lower than the T_c_ of bulk SRO, 160 K. This indicates that the SRO films were partially strained by the substrates^29^. While the residual resistivity ratio, ρ_300 K_/ρ_2 K_, of SRO(011)_pc_ of 3.6 was better than the ratio 3.0 of SRO(111)_pc_, the resistivity of SRO(011)_pc_ was much higher than that of SRO(111)_pc._ The reported residual resistivity ratios were in good agreement with other high-quality single crystalline SRO thin films by pulsed laser deposition^34,35^. Ning et al. showed that below 50 nm, the resistivity of SRO(011)_pc_ was higher than that of SRO(111)_pc_ on STO substrates because of the microstructure difference between columnar SRO(011)_pc_ and dense SRO(111)_pc_ films^36^. That can explain the higher resistivity of SRO(011)_pc_ compared to that of SRO(111)_pc_ on SAO/STO as we observed the microstructure differences in their RHEED patterns at the initial and final stages of SRO depositions (see Supplementary Figure S3) as well as their surface morphologies (see Supplementary Figure S4).

**Figure 3:**
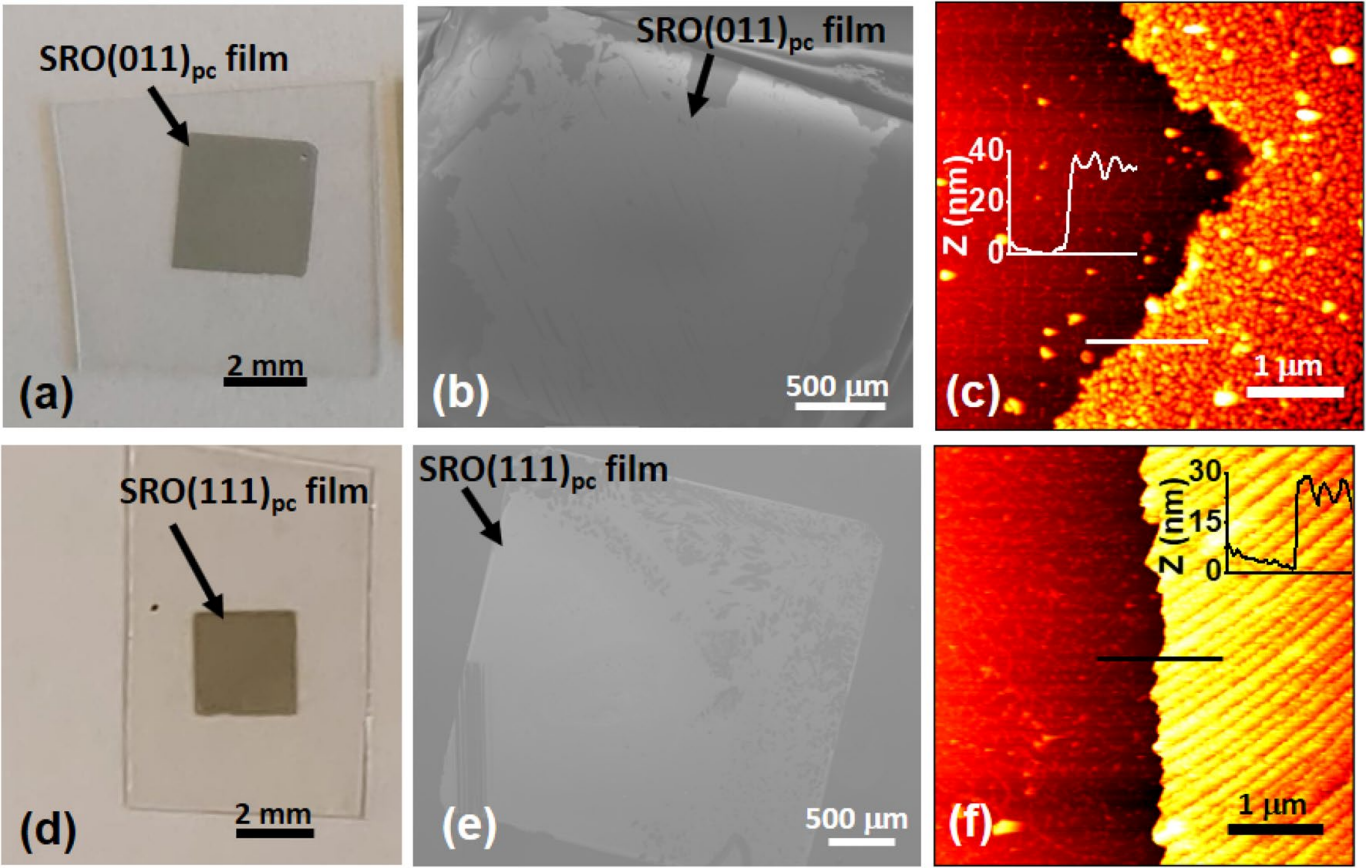
SRO(011)_pc_ and SRO(111)_pc_ films after the epitaxial lift-off and the transfer to Si(001) substrates. Millimeter-sized SRO(011)_pc_ (**a**) and SRO(111)_pc_ (**d**) films on the supporting polyethylene terephthalate (PET) substrates. SEM images of SRO(011)_pc_ (**b**) and SRO(111)_pc_ (**e**) on Si(001) substrates. Panel (**c**) and (**f**) show AFM data at the edges of SRO(011)_pc_/Si(001) and SRO(111)_pc_/Si(001), respectively. The insets show the thickness of transferred SRO films.

The SRO films were completely lifted off from the STO substrates (Fig. 3). However, the transfer process of SRO films onto Si(001) substrates resulted in cracks and some areas without SRO films. The film thicknesses were 37 nm and 24 nm for the transferred SRO(011)_pc_ and SRO(111)_pc_ films, respectively. The surface morphology of SRO films remained unchanged after the epitaxial lift-off and transfer processes (see Supplementary Figure S4).

**Figure 4:**
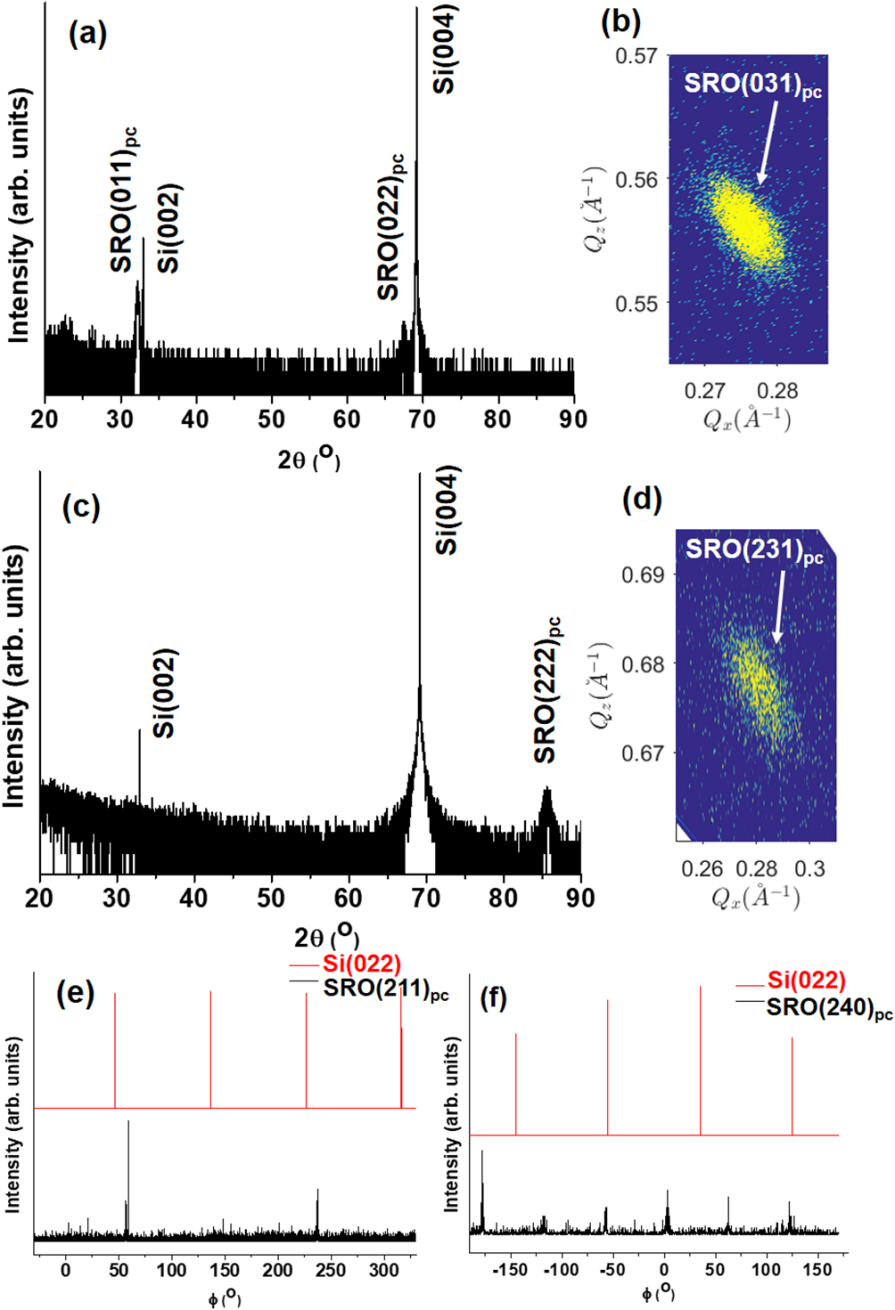
Single crystalline SRO(011)_pc_ and SRO(111)_pc_ films on Si(001). Clearly resolved reflections in 2θ-ω scans of SRO(011)_pc_ (**a**) and SRO(111)_pc_ (**c**) confirmed single oriented SRO films on Si(001) substrates. Panel (**b**) shows clear SRO(031)_pc_ reflection of SRO(011)_pc_ film in RMS, while panel (**d**) displays the SRO(231)_pc_ reflection of SRO(111)_pc_ film, which was not resolved when the film was on STO(111) substrate. The ϕ-scans reveal 2 peaks, which were separated by 180°, for the in-plane reflection SRO(211)_pc_ of SRO(011)_pc_ in panel (**e**) and 6 peaks, which were separated 60°, for the in-plane reflection SRO(240)_pc_ of SRO(111)_pc_ films in panel (**f**).

The transferred SRO(011)_pc_ on Si(001) substrates clearly showed the reflections of SRO(011)_pc_ and SRO(022)_pc_ at 32.25° and 67.49°, respectively, in the 2θ-ω scan (Fig. 4a) and the reflection of SRO(031)_pc_ in the RSM (Fig. 4b). Similarly, the SRO(222)_pc_ reflection was at 85.67° in the 2θ-ω scan (Fig. 4c) and the reflection of SRO(231)_pc_, which was not resolved in the RSM of SRO(111)_pc_/SAO(111)/STO(111), was observed in the RSM (Fig. 4d) for the transferred SRO(111)_pc_ on Si(001) thanks to the separation with the reflections of the Si(001) substrate. The lattice constants a_pc_ are 3.920 Å and 3.925 Å for transferred SRO(011)_pc_ and SRO(111)_pc_, respectively. Furthermore, Fig. 4e shows that the ϕ-scan of the in-plane reflection of SRO(211)_pc_ of the transferred SRO(011)_pc_ on Si(001) had 2 peaks, which were separated by 180°, consistent with the twofold symmetry of the SRO(011)_pc_ film. Likewise, the ϕ-scan of the in-plane reflection of SRO(240)_pc_ of the transferred SRO(111)_pc_ in Fig. 4f obtained 6 peaks, which were separated by 60° from each other, as one expected for the sixfold symmetry in the SRO(111)_pc_ film. The in-plane direction between the transferred SRO films and Si(001) substrates was not coincident, simply because the transferred SRO films were arbitrarily placed on Si(001) substrates. Therefore, the SRO films were epitaxially grown on SAO/STO with the orientation of (011)_pc_ and (111)_pc_ and remained single crystalline films after the transfer to Si(001) substrates. Freestanding single crystalline STO(011) and STO(111) films were also lifted off from SAO/STO heterostructures (see Supplementary Figure S5).

**Figure 5:**
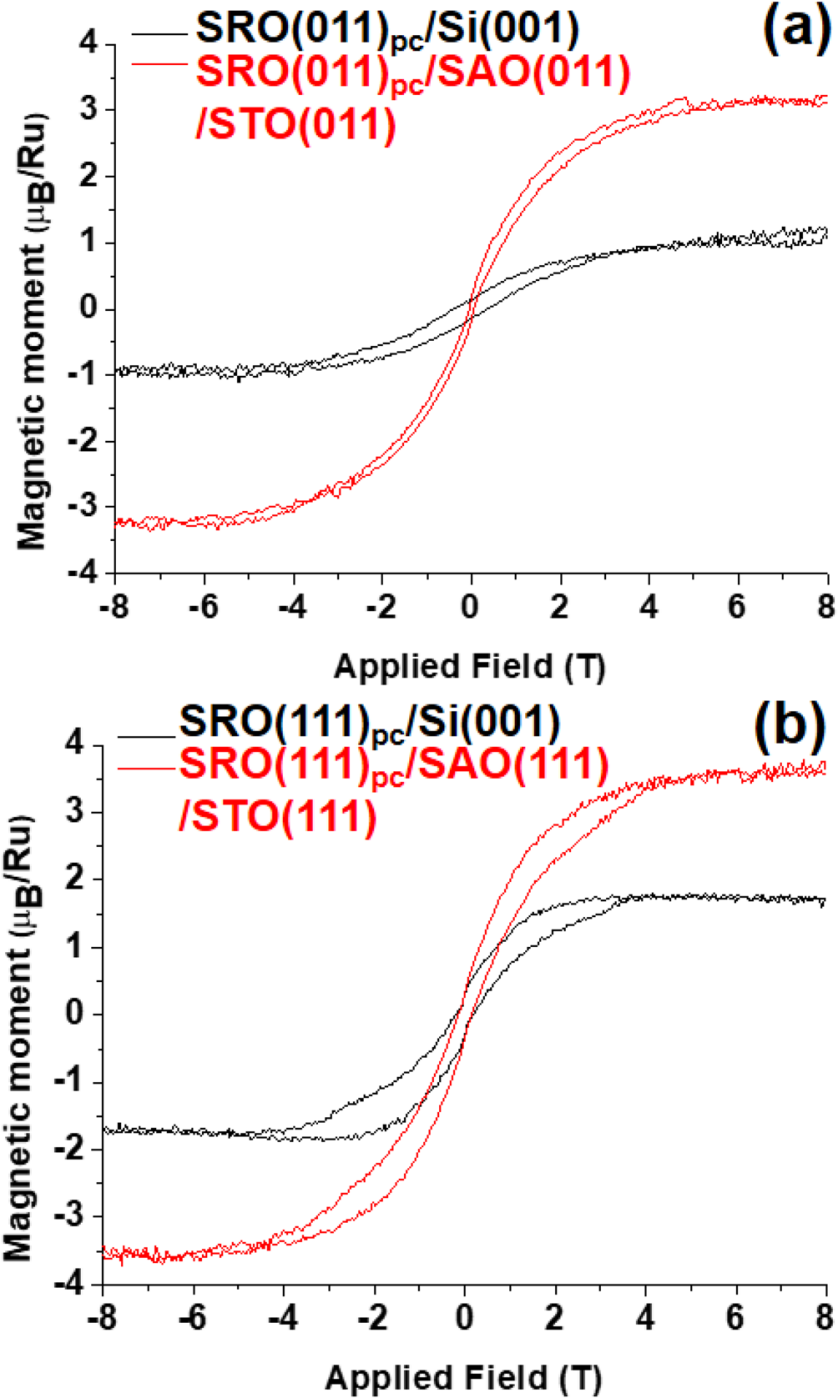
Magnetic hysteresis loops measured with the applied magnetic field perpendicular to the film surface of SRO(011)_pc_ (**a**) and SRO(111)_pc_ (**b**) films at 2 K. The saturated magnetic moment for Ru^4+^ was 3.2 μ_B_/Ru^4+^ and 3.5 μ_B_/Ru^4+^ for the SRO(011)_pc_ and SRO(111)_pc_ films, respectively, when they were on SAO/STO heterostructures, probably because of the presence of the Ru^4+^ high-spin state. After the transfer onto Si substrates, SRO(011)_pc_ and SRO(111)_pc_ films had 1.0 μ_B_/Ru^4+^ and 1.7 μ_B_/Ru^4+^, respectively, as their crystal structure were relaxed to the bulk SRO from epitaxial strain and lattice distortion by SAO/STO heterostructures.

Figure 5 shows magnetic hysteresis loops, which were measured with the applied magnetic field perpendicular to the film surface at 2 K, for SRO(011)_pc_ and SRO(111)_pc_ films before the epitaxial lift-off and after the transfer of SRO films on Si substrates. Before the epitaxial lift-off, the saturated magnetic moment was 3.2 μ_B_/Ru^4+^ and 3.5 μ_B_/Ru^4+^ for SRO(011)_pc_ and SRO(111)_pc_ films, respectively. Taken into account the change of SRO films’ volume due to the transfer of SRO films on Si, the saturated magnetic moment was 1.0 μ_B_/Ru^4+^ and 1.7 μ_B_/Ru^4+^ for SRO(011)_pc_ and SRO(111)_pc_ films, respectively. With regard to SRO, it was suggested that the low-spin Ru^4+^ state has a magnetic moment of 2 μ_B_/Ru^4+^, while the high-spin one has 4 μ_B_/Ru^4+^^34,36–40^. The bulk SRO showed 1.1–1.6 μ_B_/Ru^4+^ in the low-spin state^40^. It has been experimentally observed that the high-spin state can be stabilized using epitaxial strain and the symmetry of the lattice distortion via substrates^34,36–39^. SRO(111)_pc_ epitaxially grown on STO(111) adopted the high-spin state with the magnetic moment of 3.5–3.6 μ_B_/Ru^4+^, while SRO(011)_pc_ grown on STO(011) had a mixed state of low- and high-spin states with the magnetic moment of 3 μ_B_/Ru^4+^^36,37^. Because the epitaxial lift-off of SRO films happened in pure deionized H_2_O at room temperature, SRO films are unlikely to have undergone chemical reactions, such as acid–base and redox reactions, with H_2_O that would have affected its composition and/or structure. The magnetic moment values of SRO(111)_pc_ on SAO(111)/STO(111) suggest that the SRO(111)_pc_ film has a high-spin state, and the transferred SRO(111)_pc_ film on Si substrate adopts the low-spin state like bulk SRO, because it was not under any strain and not distorted by the weak Van der Waals bonds on the Si substrate. Similarly, the SRO(011)_pc_ films would have a mixed state of high-spin and low-spin states and the low-spin state before epitaxial lift-off and after the transfer, respectively.

## Conclusions

In this study, we have shown freestanding single crystalline of SrRuO_3_ films with (011) and (111) orientations were successfully synthesized using water-sacrificial SAO layers on single crystal STO substrates. The water-sacrificial SAO layers were epitaxially grown on STO(011) and STO(111) because of lattice mismatch of 1.43% and similar 2D symmetry of crystal planes. The single crystalline (011)_pc_- and (111)_pc_-oriented SRO films were successfully transferred on Si substrates. SRO films on SAO(011)/STO(011) and SAO(111)/STO(111) heterostructures showed enhanced magnetism of the high-spin state, whereas the transferred SRO films on Si showed bulk-like magnetism of the low-spin state. This study demonstrates the possibility to obtain single crystalline oxide perovskite films with desirable orientation on arbitrary substrates.

## Methods

Well-defined atomically flat STO substrates were obtained by treating STO with buffered hydrogen fluoride solution and then thermal annealing as described elsewhere^41,42^. PLD was performed in a vacuum system with a base pressure of 2 × 10^−8^ mbar, equipped with an in situ RHEED and a KrF excimer laser of 248 nm (COMPexPro from Coherent Inc.). The central part of the laser beam was selected with a mask and focused on polycrystalline SAO, SRO, and single crystal STO targets. The substrate temperature 700 °C, laser repletion rate 1 Hz, spot size 1.8 mm^2^ and substrate-target distance 50 mm were the same for the growth of SAO, SRO and STO layers. Laser energy density and oxygen pressure were 1.25 J cm^−2^ and 10^−3^ mbar for SAO, 1.3 J cm^−2^ and 10^−2^ mbar for STO, and 2.1 J cm^−2^ and 8 × 10^−3^ mbar for SRO. After deposition, the samples were cooled down to room temperature at a maximum rate of 20 °C min^−1^ at the deposition pressure for STO/SAO/STO heterostructures and at 100 mbar oxygen pressure for SRO/SAO/STO heterostructures. The growth rate was 1.88 nm per 100 pulses for SAO(011) and SAO(111) (see Supplementary Figure S6). The transfer of epitaxial films from SAO water-sacrificial layers on supporting substrates was done as described elsewhere^17,43^.

The crystal structure of samples was analyzed using PANalytical X’Pert Pro with the Inc. beam Monochr. 4xGe220 Cu asym. LF monochromator to select Cu Kα_1_ radiation. Scanning electron microscope (SEM) (Jeol JSM-6490) was used to acquire the images of the transferred SRO films on Si substrates in order to calculate the transferred film area based on the contrast between SRO and Si. Surface morphologies of samples were investigated using atomic force microscopy (AFM), Bruker Dimension ICON, operating in tapping mode and analyzed by Gwyddion software^44^. The transport measurements were performed in the four-probe Van der Pauw configuration and the magnetic properties were measured with the magnetic field perpendicular to the film surface using vibrating sample magnetometry in a Quantum Design Physical Properties Measurement System.

## Supplementary Information

Supplementary Information contains X-ray reflectivity, atomic force microscopy data and X-ray diffraction of heterostructures of SrRuO_3_/Sr_3_Al_2_O_6_/SrTiO_3_ and freestanding SrTiO_3_ films.

Supplementary Information.
