# Supplementary material for: Epitaxial lift-off of freestanding (011) and (111) SrRuO3 thin films using a water sacrificial layer
Source: Sci Rep. 2021 Jun 14;11:12435. doi: 10.1038/s41598-021-91848-2 (PMC8203781; doi:10.1038/s41598-021-91848-2)
Supplement: Supplementary file 1 — Supplementary Information. [file 41598_2021_91848_MOESM1_ESM.docx]

Supplementary information

Epitaxial Lift-off of Freestanding (011) and (111) SrRuO_3_ Thin Films Using Water Sacrificial Layer

Phu T.P. Le, Johan E. ten Elshof and Gertjan Koster*

MESA+ Institute for Nanotechnology, University of Twente, P.O. Box 217, 7500 AE Enschede, The Netherlands

Corresponding author

*Email address: g.koster@utwente.nl


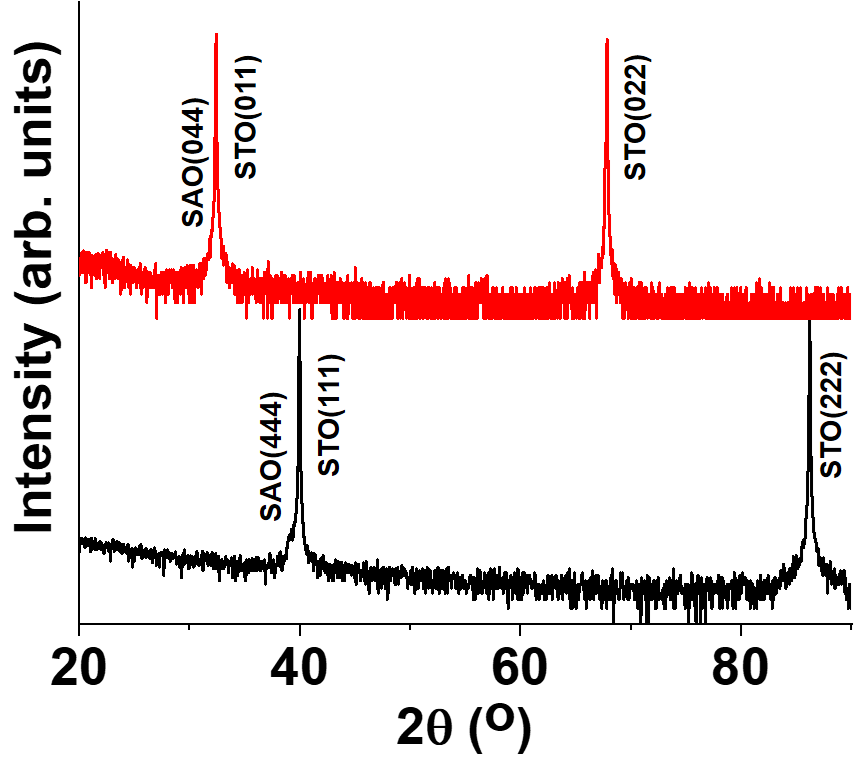


**Supplementary Figure S1.** X-ray diffraction (XRD) patterns of 100 nm SAO(011) and 100 nm SAO(111) on STO substrates with the capping STO layers. There is no other phases and other orientations of SAO except the desired SAO(011) and SAO(111) orientations, which are hard to be observed in these full scans.


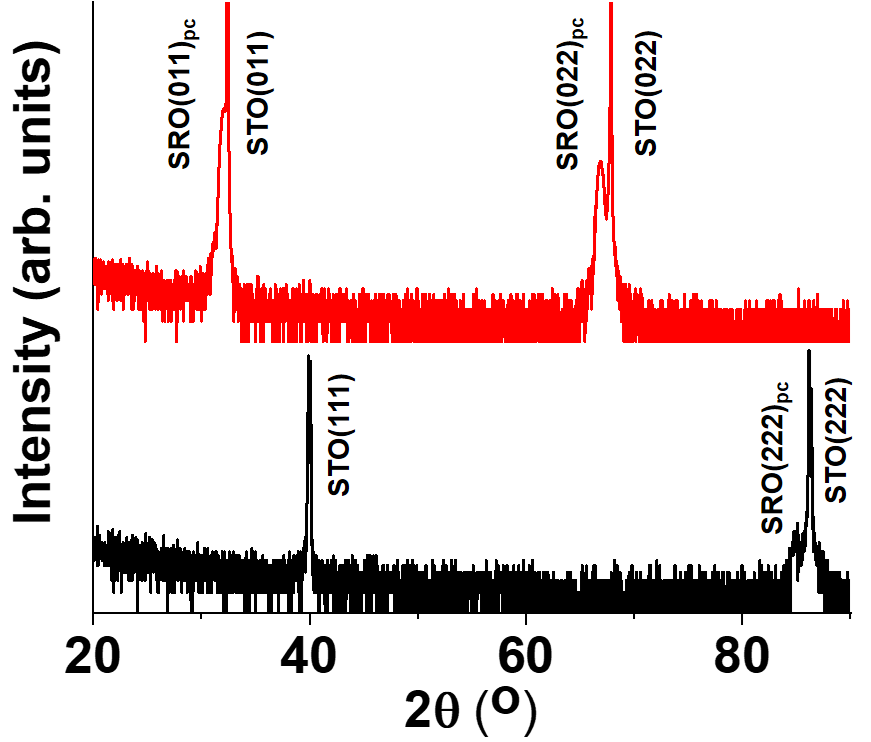


**Supplementary Figure S2.** XRD patterns of SRO(011)_pc_/STO(011) and SRO(111)_pc_/STO(111), which were deposited under the same growth condition of SRO films on SAO/STO samples.


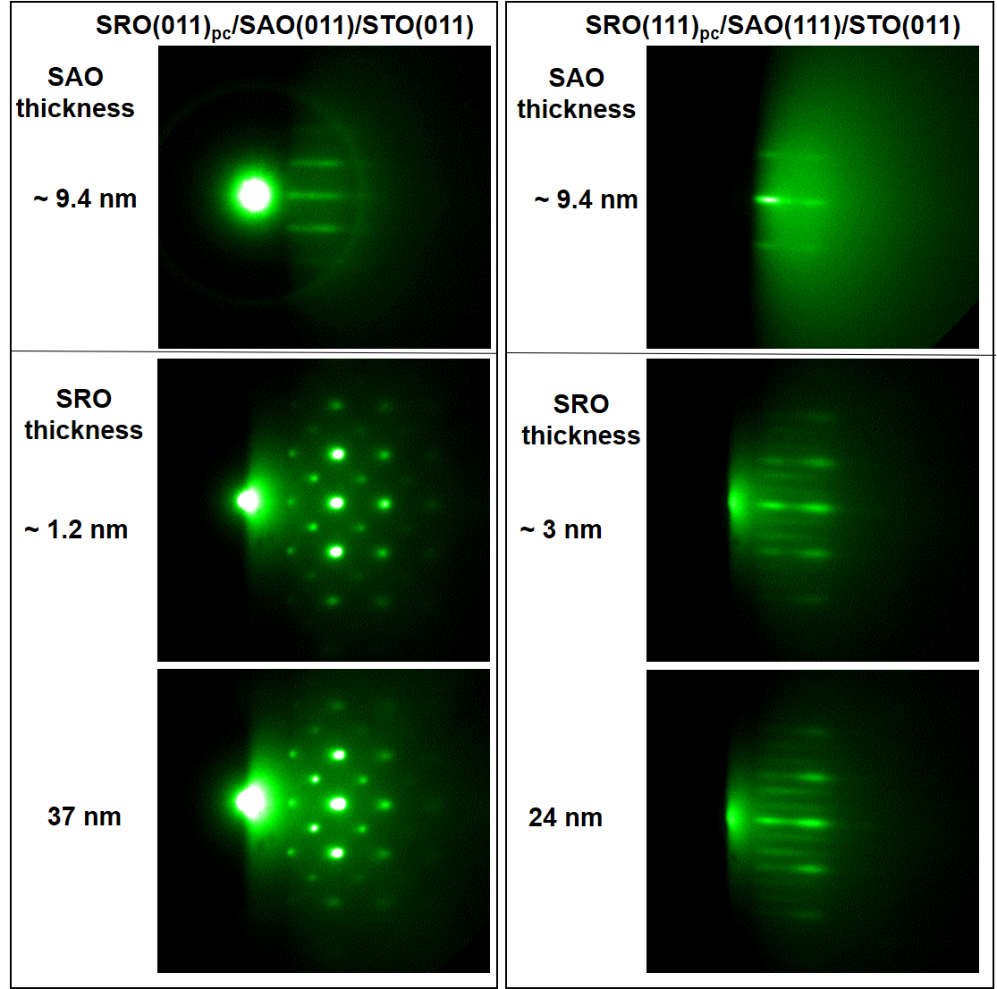


**Supplementary Figure S3.** RHEED patterns of SAO(011), SAO(111), SRO(011)_pc_ and SRO(111)_pc_ of SRO/SAO/STO heterostructures at different stages during PLD. The growth mode of SRO layers remained almost unchanged during the deposition. SRO was grown on SAO(011)/STO(011) in the 3D growth mode with the formation of nanoscale islands, while it was grown smoother on SAO(111)/STO(111). The RHEED patterns of 37 nm SRO(011)_pc_/SAO(011)/STO(011) and 24 nm SRO(111)_pc_/SAO(111)/STO(111) were from the insets of Figure 2a and 2b.


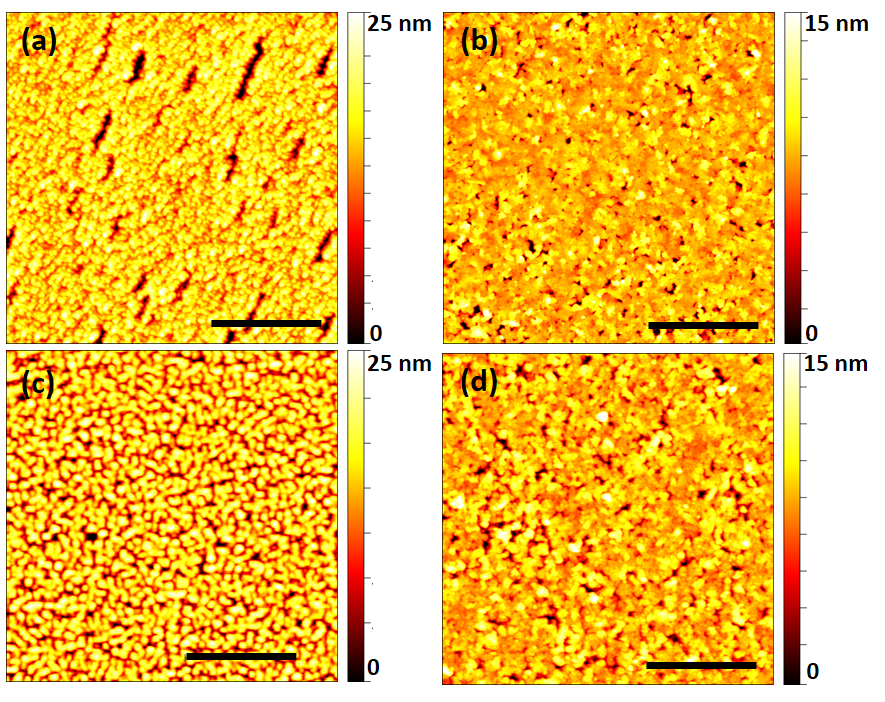


**Supplementary Figure S4**. AFM data of SRO(011)_pc_ and SRO(111)_pc_ films before the epitaxial lift-off and after the transfer on Si(001) substrates. Panel (a) and (b) show the surface morphology of SRO films in SRO(011)_pc_/SAO(011)/STO(011) and SRO(111)_pc_/SAO(111)/STO(111) with RMS roughness of 3.4 nm and 1.9 nm, respectively. Panel (c) and (d) display the surface morphology of SRO(011)_pc_ and SRO(111)_pc_ on Si(001) substrates with RMS roughness of 6.8 nm and 2.2 nm, respectively. The scale bar is 1 μm.


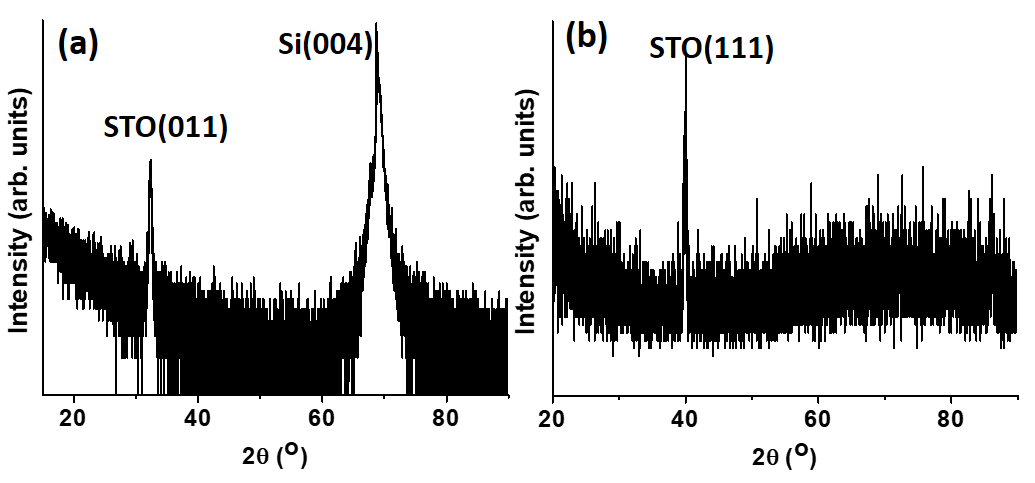


**Supplementary Figure S5.** XRD patterns of STO films lifted off from SAO/STO heterostructures, STO(011) on Si(001) (a) and STO(111) on polydimethylsiloxane (PDMS).


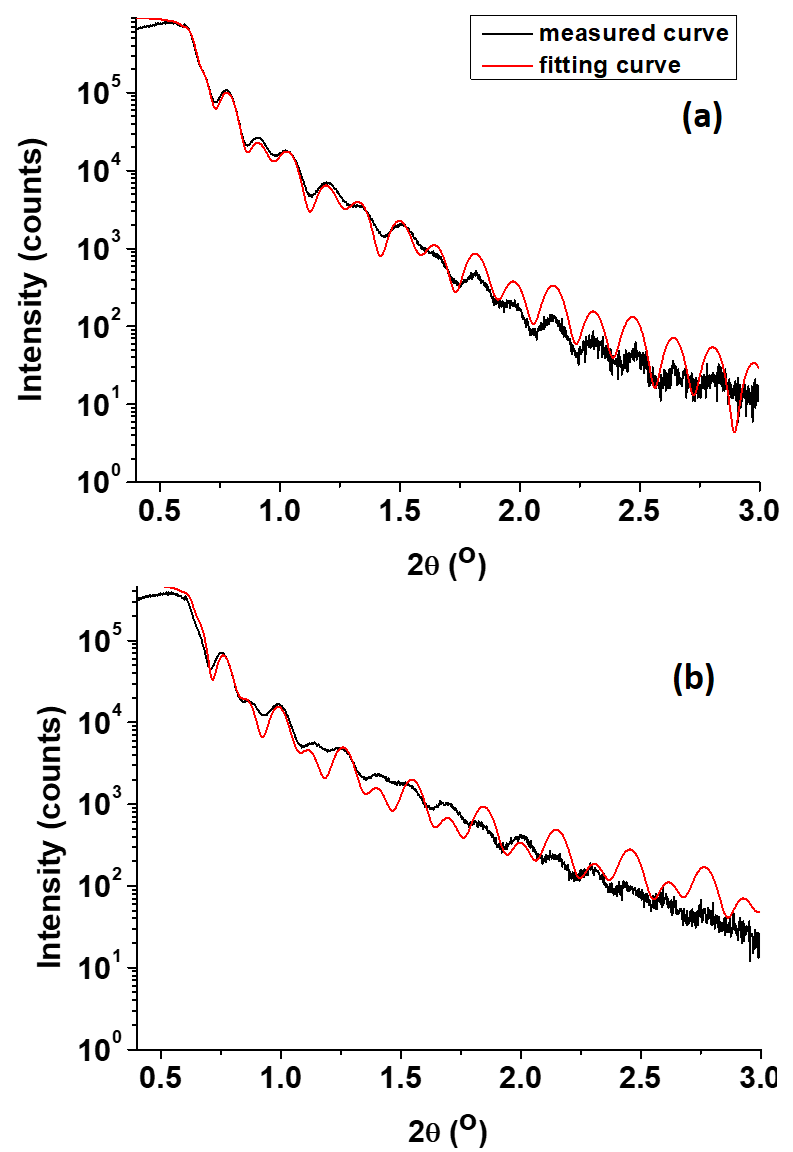


**Supplementary Figure S6**. X-ray reflectivity curves and their fitting curve, which were obtained by X’Pert Reflectivity software, of STO(011)/SAO(011)/STO(011) (a) and STO(111)/SAO(111)/STO(111) (b) heterostructures. The thickness was 23.5 nm for 1250 pulses of SAO(011) and 27.4 nm for 1450 pulses of SAO(111). The STO thickness was 28 nm for both samples.
